# The dynamics of cortical GABA in human motor learning

**DOI:** 10.1113/JP276626

**Published:** 2018-11-02

**Authors:** James Kolasinski, Emily L. Hinson, Amir P. Divanbeighi Zand, Assen Rizov, Uzay E. Emir, Charlotte J. Stagg

**Affiliations:** ^1^ Wellcome Centre for Integrative Neuroimaging Oxford Centre for fMRI of the Brain Nuffield Department of Clinical Neurosciences University of Oxford Oxford OX3 7DU UK; ^2^ Cardiff University Brain Research Imaging Centre School of Psychology Cardiff University Maindy Road Cardiff CF24 4HQ UK; ^3^ Oxford Centre for Human Brain Activity Wellcome Centre for Integrative Neuroimaging Department of Psychiatry University of Oxford Oxford OX3 7JX UK; ^4^ Purdue University School of Health Sciences 550 Stadium Mall Drive West Lafayette IN 47907 USA

**Keywords:** GABA, Motor cortex, Plasticity

## Abstract

**Key points:**

- The ability to learn new motor skills is supported by plasticity in the structural and functional organisation of the primary motor cortex in the human brain.
- Changes inhibitory to signalling by GABA are thought to be crucial in inducing motor cortex plasticity.
- This study used magnetic resonance spectroscopy (MRS) to quantify the concentration of GABA in human motor cortex during a period of motor learning, as well as during a period of movement and a period at rest.
- We report evidence for a reduction in the MRS‐measured concentration of GABA specific to learning. Further, the GABA concentration early in the learning task was strongly correlated with the magnitude of subsequent learning: higher GABA concentrations were associated with poorer learning.
- The results provide initial insight into the neurochemical correlates of cortical plasticity associated with motor learning, specifically relevant in therapeutic efforts to induce cortical plasticity during recovery from stroke.

**Abstract:**

The ability to learn novel motor skills is a central part of our daily lives and can provide a model for rehabilitation after a stroke. However, there are still fundamental gaps in our understanding of the physiological mechanisms that underpin human motor plasticity. The acquisition of new motor skills is dependent on changes in local circuitry within the primary motor cortex (M1). This reorganisation has been hypothesised to be facilitated by a decrease in local inhibition via modulation of the neurotransmitter GABA, but this link has not been conclusively demonstrated in humans. Here, we used 7 T magnetic resonance spectroscopy to investigate the dynamics of GABA concentrations in human M1 during the learning of an explicit, serial reaction time task. We observed a significant reduction in GABA concentration during motor learning that was not seen in an equivalent motor task lacking a learnable sequence, nor during a passive resting task of the same duration. No change in glutamate was observed in any group. Furthermore, M1 GABA measured early in task performance was strongly correlated with the degree of subsequent learning, such that greater inhibition was associated with poorer subsequent learning. This result suggests that higher levels of cortical inhibition may present a barrier that must be surmounted in order to achieve an increase in M1 excitability, and hence encoding of a new motor skill. These results provide strong support for the mechanistic role of GABAergic inhibition in motor plasticity, raising questions regarding the link between population variability in motor learning and GABA metabolism in the brain.

## Key points

- The ability to learn new motor skills is supported by plasticity in the structural and functional organisation of the primary motor cortex in the human brain.
- Changes inhibitory to signalling by GABA are thought to be crucial in inducing motor cortex plasticity.
- This study used magnetic resonance spectroscopy (MRS) to quantify the concentration of GABA in human motor cortex during a period of motor learning, as well as during a period of movement and a period at rest.
- We report evidence for a reduction in the MRS‐measured concentration of GABA specific to learning. Further, the GABA concentration early in the learning task was strongly correlated with the magnitude of subsequent learning: higher GABA concentrations were associated with poorer learning.
- The results provide initial insight into the neurochemical correlates of cortical plasticity associated with motor learning, specifically relevant in therapeutic efforts to induce cortical plasticity during recovery from stroke.

## Introduction

Motor learning describes the process by which we change and adapt in our interactions with the external world (Dayan & Cohen, 2011). The ability to acquire new motor skills has been strongly associated with plastic changes in both the structural and the functional organisation of the primary motor cortex (M1) (Dayan & Cohen, 2011; Sampaio‐Baptista *et al*. 2018). Specifically, evidence from both human and non‐human primate studies suggests that repeated practice of a motor skill is associated with changes in the topographic organisation of the region (Nudo *et al*. 1996; Karni *et al*. 1998). Further, the learning of fine motor skills has been associated with synaptogenesis in M1 (Kleim *et al*. 2002), as well as changes in the myelination of the underlying white matter (Sampaio‐Baptista *et al*. 2013). Understanding the physiological processes that drive the observed structural and functional changes in M1 to support motor learning are necessary for the development of therapeutic approaches to promote adaptive plasticity after brain injuries, such as stroke, via facilitation of the re‐learning of motor skills compromised by brain pathology.

There is a strong body of evidence to suggest that a reduction in cortical inhibitory tone is critical for the induction of M1 plasticity (Bachtiar & Stagg, 2014; Peters *et al*. 2017). Specifically, a reduction in GABAergic signalling appears crucial to the induction of long‐term potentiation (LTP)‐like plasticity in M1 (Castro‐Alamancos *et al*. 1995; Trepel & Racine, 2000), potentially by unmasking or potentiating latent pre‐existing horizontal connections in the cortex (Huntley, 1997). In addition, particularly compelling evidence for the role of GABAergic disinhibition in promoting M1 plasticity is provided by recent work using *in vivo* two‐photon imaging in mouse M1 (Chen *et al*. 2015). In their study, learning resulted in a significant reduction in axonal boutons observed on somatostatin‐expressing inhibitory neurons. Optogenetically manipulating activity in this inhibitory neuronal population during learning both disrupted the observed dendritic structural changes and affected motor performance.

Evidence of disinhibition in human M1 mediated via the GABAergic system has been reported during and following motor learning using transcranial magnetic stimulation (TMS) (Rosenkranz *et al*. 2007; Coxon *et al*. 2014). The responsiveness of the GABAergic system to transcranial direction current stimulation (tDCS) has been correlated with the magnitude of motor learning in healthy control participants (Stagg *et al*. 2011 *a*). Evidence for a reduction in cortical GABA has also previously been reported in the context of motor learning over the course of weeks, with correlated changes in the strength of connectivity in the resting state motor network as a whole (Sampaio‐Baptista *et al*. 2015). Lower levels of M1 GABA have also been reported in the chronic stages of recovery after stroke, relative to unaffected individuals. In these patients a reduction in M1 GABA is associated with a favourable clinical response to a therapeutic intervention (Blicher *et al*. 2015). However, using magnetic resonance spectroscopy (MRS), only one study to date has reported direct evidence for a reduction in M1 GABA in humans during the learning of a new motor skill (Floyer‐Lea, 2006); a result which has proven difficult to replicate.

**Figure 1:**
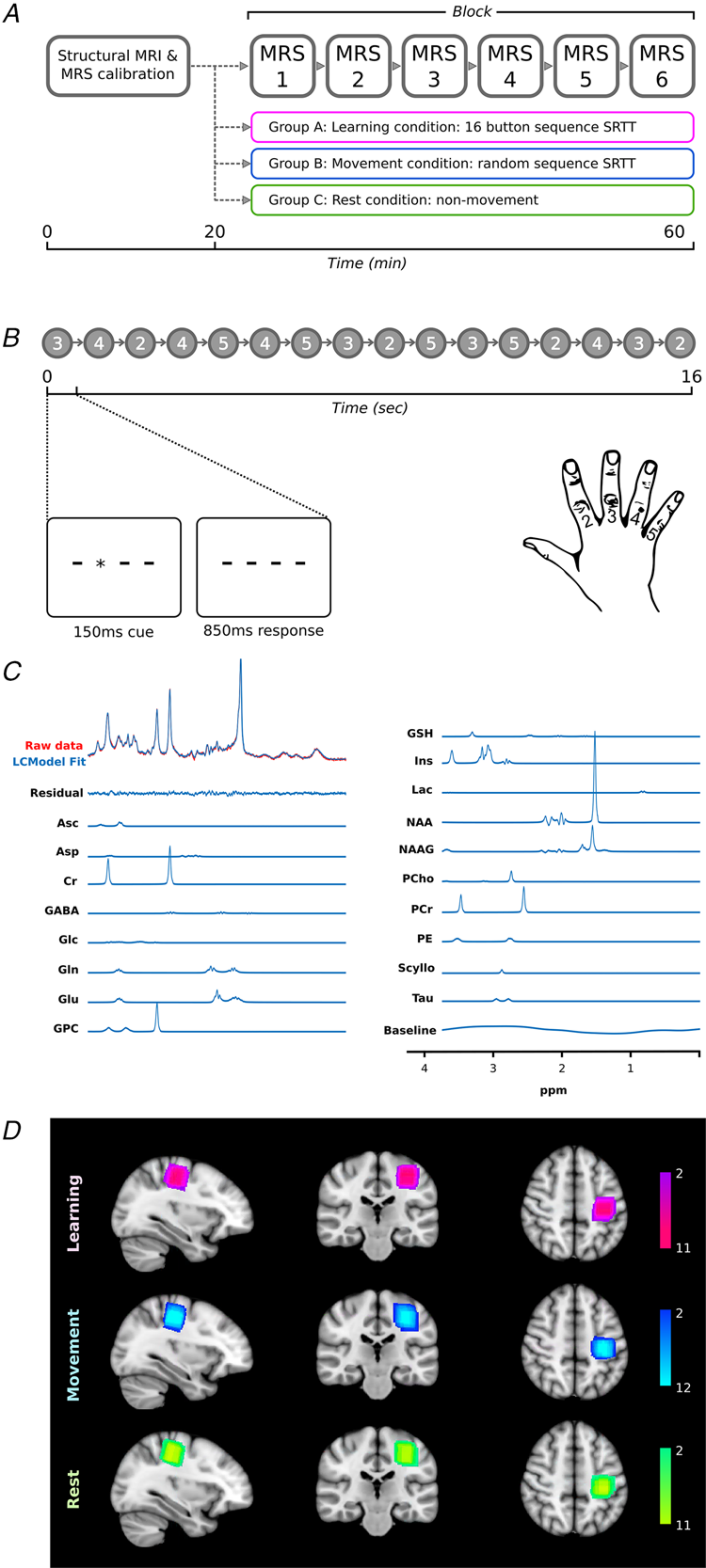
Experimental design and MRS data acquisition *A*, MRS data were acquired in six independent blocks during a concurrent task which differed across the three experimental groups: the Learning group performed a 16‐button press, repeating serial reaction time task; the Movement group performed a serial reaction time task without a repeating sequence; the Rest group passively observed a video. *B*, the Learning group performed the same 16‐button press sequence with three repeats per epoch (48 s), with each epoch separated by a 12 s rest period. MRS data were acquired in each block (64 averages) and analysed using LCModel (Provencher, 2001). *C*, representative acquisition from one participant in one M1 block, including model fit. *D*, M1 mRS voxels were centred over the left (contralateral) hand knob illustrated in as heatmaps in the three experimental groups: colour bars represent the number of participants.

Here, we take advantage of the increased spatial, temporal and spectral resolution afforded by acquiring MRS data at ultra‐high field (7 T) to investigate the changes in M1 GABA during motor learning (Fig. 1). We sought to address the hypothesis that MRS‐assessed M1 GABA will decrease during learning of a motor task, and further that GABA concentration early in learning will predict the magnitude of subsequent motor learning (Stagg *et al*. 2011).

## Materials and methods

### Ethical approval

All participants gave written informed consent to participate in the study. This study was subject to ethical review and approval from Oxford University Central Research Ethics Committee Approval (MSD‐IDREC‐C1‐2014‐100). The study conformed to the standards set by the *Declaration of Helsinki*, except for registration in a database.

### Participants

Fifty‐one participants were recruited to the study. Participants were right handed according to the Edinburgh Handedness Questionnaire (Oldfield, 1971) and met local safety criteria for scan participation at 7 T. Participants with high musical ability, defined as Grade 6 or above by the Associated Board of the Royal Schools of Music criteria, were excluded. Participants were allocated to one of three experimental groups: Learning, Movement or Rest (Fig. 1 *A*). A full breakdown of group allocation including age and gender is provided in Table 1. No participant was recruited to more than one group and groups were matched as far as possible for age and sex. All participants attended one scanning session.

**Table 1:** Participant breakdown across experimental groups

	Learning	Movement	Rest
Participants recruited	18	19	14
Excluded: CRLB	6	7	2
Excluded: Grubb's test MRS	0	0	1
Excluded: Grubb's test reaction time	1	0	0
Total usable participants	11	12	11
Age (years, mean ± SD)	24.2 ± 3.7	24.6 ± 4.1	23.3 ± 4.8
Gender	7 Female	6 Female	5 Female

### MR data acquisition

Magnetic resonance imaging (MRI) and MRS data were acquired using a 7 T Siemens Magnetom System (Siemens, Erlangen, Germany) with a 32‐channel head coil (Nova Medical Inc., Wilmington, MA, USA). A dielectric pad (barium titanate: 0.5 × 11 × 11 cm) was positioned dorsally on the scalp over left central sulcus to increase B1 efficiency in the M1 voxel of interest (VOI) (Lemke *et al*. 2015). B1 efficiency was imaged using actual flip angle imaging (AFI): field of view 240 × 240, TR1 6 ms, TR2 30 ms, TE 2.58 ms, non‐selective flip angle 60°, slice thickness 2.5 mm.

To enable placement of the MRS voxel, structural MRI data were acquired with a Magnetization Prepared Rapid Acquisition Gradient Echo (MPRAGE) sequence: TR = 2200 ms, TE = 2.82 ms, slice thickness 1.0 mm, in‐plane resolution 1.0 × 1.0 mm, GRAPPA factor = 2.

MRS data were acquired using a semi‐LASER sequence (localisation by adiabatic selective refocusing) (van de Bank *et al*. 2015): TR = 5000 ms, TE = 36 ms, 20 × 20 × 20 mm voxel, 64 averages per block, TA = 5 min 20 s, using VAPOR (VAriable Power RF pulses with Optimized Relaxation delays) water suppression (Tkác *et al*. 1999). The VOI was manually positioned in the left M1, covering the whole hand knob (Yousry *et al*. 1997) (Fig. 1 *D*) and excluding the dura. MRS data were acquired in six blocks of approximately 5 min each (Fig. 1 *A*). During the acquisition of the MRS data, participants either performed an explicit sequence learning task, performed a motor task without a learnable sequence or watched a video.

### MRS task stimuli

Both the Learning and Movement groups were engaged in a visually cued serial reaction time task (SRTT). Responses were made with the right hand via a four‐button button box resting on the participant's thigh. Visual cues consisted of four horizontal lines displayed on a screen, representing the four buttons (Fig. 1 *B*). Each cue consisted of one line being replaced with an asterisk for 150 ms. Participants were instructed to respond by pressing the corresponding button as quickly and accurately as possible, and not to press in anticipation of an upcoming cue. There was an inter‐stimulus interval (ISI) of 850 ms between cues. In total, 48 cues were presented in each epoch, followed by a rest period of 12 s. The task repeated throughout MRS acquisition (Fig. 1 *A*).

In the Learning group participants were explicitly informed to expect a repeating sequence in the cues (Fig. 1 *B*: a 16‐item sequence repeated three times per epoch). In the Movement group, participants were told not to expect a sequence; cues were pseudo‐randomised to produce different sequences of 48 cues in each epoch, and the number of button presses for each finger was matched to the learning task.

In the Rest group, participants watched a 40 min excerpt from a nature documentary. Between each MRS block, participants were cued to press a button.

### MR data analysis

MRS data from each block were corrected using an unsuppressed water signal acquired from the same VOI in the single acquisition following the acquisition of the six serial blocks of MRS data. MRS data were then subject to eddy current correction and a zero‐order phasing of array coil spectra using in‐house scripts. Any residual water signal was removed using Hankel–Lanczos singular value decomposition (Cabanes *et al*. 2001). LCModel analysis was used to quantify a concentration of neurochemicals within the chemical shift range 0.5–4.2 ppm (Provencher, 2001). The exclusion criteria for data were as follows: Cramér–Rao bounds (CRLB) > 50%, water linewidths at full width at half maximum (FWHM) > 15 Hz or signal/noise ratio (SNR) < 30. There was no strong correlation (> ±0.3) between GABA and other metabolites, indicating good spectral separation was achieved. A breakdown of MRS quality metrics across experimental condition and block are provided in Table 2.

**Table 2:** Breakdown of MRS data quality metrics by group and by block

	SNR	CRLB (GABA)	FWHM	Fit
*Learning*	*53.3 ± 5.4*	*26.2 ± 10.3*	*10.2 ± 2.2*	*1168.9 ± 112.7*
Block 1	53.7 ± 7.6	23.1 ± 9.5	9.9 ± 2.2	1147.5 ± 59.9
Block 2	51.6 ± 10.2	23.1 ± 10.1	10.2 ± 2.0	1175.4 ± 106.0
Block 3	51.7 ± 11.3	24.1 ± 8.3	10.3 ± 2.3	1188.0 ± 137.0
Block 4	52.2 ± 10.9	27.5 ± 11.7	10.4 ± 2.4	1164.4 ± 120.5
Block 5	52.1 ± 10.2	26.1 ± 10.2	10.2 ± 2.6	1160.8 ± 125.7
Block 6	50.9 ± 12.4	29.9 ± 10.3	10.2 ± 2.2	1177.3 ± 132.4
*Movement*	*53.1 ± 5.6*	*27.8 ± 6.9*	*9.2 ± 1.4*	*1115.3 ± 83.9*
Block 1	54.3 ± 3.4	29 ± 8.7	9.0 ± 1.3	1129.9 ± 65.6
Block 2	54.8 ± 4.4	29 ± 8.3	9.3 ± 1.8	1107.8 ± 76.5
Block 3	54.9 ± 3.6	31.6 ± 6.9	9.1 ± 1.7	1108.7 ± 104.5
Block 4	53.8 ± 3.4	29.3 ± 5.9	8.9 ± 1.4	1125.7 ± 82.7
Block 5	54.2 ± 4.0	26.6 ± 4.4	9.4 ± 1.4	1099.2 ± 89.2
Block 6	53.0 ± 3.0	27.3 ± 7.1	9.2 ± 1.2	1120.1 ± 93.9
*Rest*	*53.2 ± 5.4*	*26.5 ± 7.8*	*9.2 ± 1.5*	*1099.1 ± 103.4*
Block 1	53.2 ± 6.1	27.3 ± 7.4	9.3 ± 2.0	1098.0 ± 84.7
Block 2	51.9 ± 6.3	27.0 ± 7.7	9.1 ± 2.1	1114.1 ± 106.7
Block 3	52.3 ± 4.7	27.8 ± 9.2	9.1 ± 1.2	1091.6 ± 87.3
Block 4	51.8 ± 4.1	26.6 ± 9.5	9.5 ± 1.1	1106.6 ± 106.2
Block 5	52.3 ± 6.5	27.1 ± 9.6	9.3 ± 1.5	1103.2 ± 142.0
Block 6	53.1 ± 4.9	27.4 ± 10.1	8.8 ± 1.0	1081.0 ± 105.4
*Statistics*				
Interaction: Group × Time	*F*_(10,155)_ = 0.725,	*F*_(10,155)_ = 1.252,	*F*_(10,155)_ = 0.800,	*F*_(10,155)_ = 0.784,
	*p* = 0.700	*p* = 0.262	*p* = 0.629	*p* = 0.645
Main effect: Time	*F*_(5,155)_ = 0.769,	*F*_(5,155)_ = 0.573,	*F*_(5,155)_ = 0.496,	*F*_(5,155)_ = 0.223,
	*p* = 0.573	*p* = 0.721	*p* = 0.779	*p* = 0.952

SNR: signal/noise ratio; CRLB: Cramér–Rao bounds; FWHM: full‐width half maximum; Fit: area under curve from rectified residuals of LCmodel fit. Values are shown as mean ± SD.

To quantify the proportion of white matter (WM), grey matter (GM) and cerebrospinal fluid (CSF) in the VOI, FMRIB's Automated Segmentation Tool (FAST) (Zhang *et al*. 2001) was applied to the T1‐weighted MPRAGE scan. GABA and glutamate peaks were corrected for the proportion of GM in the VOI. Total creatine (including phosphocreatine: tCr) peaks were corrected for the proportion of total brain tissue in the VOI.

MRS data analysis therefore yielded independent quantification of neurochemical concentrations corresponding to each of the six MRS blocks, expressed as a ratio of tCR, for example GABA:tCr and Glu:tCr (Fig. 1 *A*).

### MRS motor task data analysis

In the Learning and Movement groups, learning was assessed by quantifying response time (RT) between stimulus presentation and button press response for correct responses only. RT data were divided into blocks corresponding to the six independent MRS acquisitions. Median RT was then calculated within each block for each participant and used for subsequent analysis. A median RT from blocks 4–6 was calculated for each subject and this was used to calculate a percentage change from the median RT in the first sequence block to give a measure of motor learning, i.e. [(*RT* (4−6) − *RT* (1))/*RT* (1)] (Stagg *et al*. 2011 *a*). In addition, a secondary measure of best block performance was calculated, defined as the difference between the block with the lowest median RT and the first block, expressed as a percentage change from the median RT in the first sequence block, (i.e. (*RT* (*Best*) − *RT* (1))/*RT* (1)]. A measure of task accuracy was made, defined as the number of correct button presses made per block.

### Statistics

Statistical analyses and graphing were conducted using JMP (Version 13.0, SAS Institute, Cary, NC, USA) and Statistics Package for the Social Sciences (SPSS, Version 22.0, IBM Corp., Armork, NY, USA). To compare changes in RT across the Learning and Movement groups, median RT data for each block were subject to a two‐way mixed ANOVA, with experimental group (Learning or Movement) as the between‐subjects factor, and time (block 1–6) as the within‐subjects factor. Changes in task accuracy were assessed using an analogous approach

To make similar comparisons regarding changes in the MRS concentration of GABA and glutamate across the three experimental groups, GM‐corrected GABA:tCr and Glu:tCr values were also separately subject to a two‐way mixed ANOVA, with experimental group (Learning, Movement, Rest) as the between subjects factor, and time (blocks 1–6) as the within‐subjects factor. The mixed ANOVAs performed on MRS data were conducted using raw non‐normalised data; for visualisation purposes only, data in Fig. 3 were normalised to block 1. Significant interactions were followed up with analysis of simple main effects within each experimental group. Correlations were assessed using Pearson's correlation coefficients (two‐tailed). Comparison of correlation statistics was undertaken using Hittner's test (Hittner *et al*. 2003). A total of 17 participants were excluded on the basis of pre‐defined criteria (Table 1): 15 participants were excluded on the basis of their MRS CRLB exceeding 50%; one participant was excluded from the Rest group due to a statistical outlier in their GABA:tCr values (criteria: ±2 SD; participant data above mean in block 5); and one participant was excluded from the Learning group due to a statistical outlier in the median RT values (criteria: ±2 SD; participant data above mean in block 4). Normality of the remaining data was confirmed using Shapiro–Wilk tests.

## Results

### Motor sequence learning is associated with a reduction in M1 GABA concentration

We observed a significant reduction in MRS measures of M1 GABA over time in the Learning group, in line with the significant reduction in RT over time in this group. No change was observed in RT in the Movement group, and no reduction in GABA:tCr was observed in either the Movement group or the Rest group.

**Figure 2:**
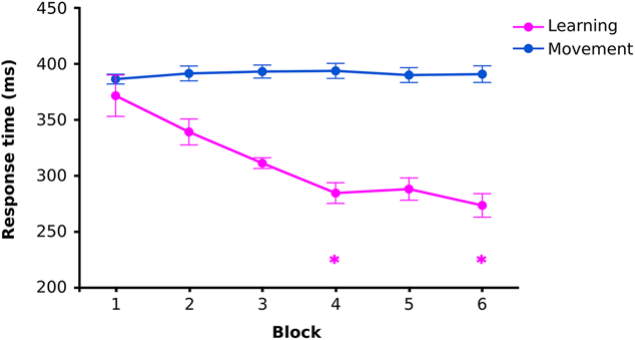
Learning of motor sequence serial reaction time task Group mean response time data showing a decrease in response times in the Learning SRTT as the participants learned the four‐button 16‐press sequence (magenta). No equivalent learning was observed in the Movement group SRTT of equivalent duration, which contained no repeating sequence (blue). Two‐way mixed ANOVA: experimental group (Learning or Movement) as between‐subjects factor and time (blocks 1–6) as within‐subjects factor: *F*_(1.49,31.24)_ = 10.52, *p* = 0.001, partial η^2^ = 0.334 (Greenhouse–Geisser corrected). This effect was driven by a reduction in response time in the Learning group (simple main effect of block: *F*_(1.4,14.0)_ = 9.33, *p* = 0.005, partial η^2^ = 0.483, Greenhouse–Geisser corrected). ^*^ *p* < 0.05 Bonferroni‐adjusted *post hoc* pairwise comparison compared with block 1. Within‐subject standard error bars calculated across each group (Cousineau, 2005).

In the analysis of RT data, a two‐way mixed ANOVA revealed a significant interaction between experimental group (Learning or Movement) and time on RT: *F*_(1.49,31.24)_ = 10.52, *p* = 0.001, partial η^2^ = 0.334 (Greenhouse Geisser corrected), and therefore simple main effects were analysed (Fig. 2). In the Learning group there was a significant main effect of time on RT: *F*_(1.41,14.0)_ = 9.33, *p* = 0.005, partial η^2^ = 0.483 (Greenhouse Geisser corrected). *Post hoc* paired‐samples *t* tests revealed a significant reduction in RT in the Learning group in blocks 4 and 6 compared with block 1 (*p* < 0.05, Bonferroni adjusted). In the Movement group there was no significant main effect of time on RT: *F*_(5,55)_ = 0.89, *p* = 0.496, partial η^2^ = 0.075.

We observed no significant difference in task accuracy comparing the first block of the learning and movement tasks (independent samples *t* test *t*(21) = −0.670, *p* = 0.510). Further, there was no significant change in task accuracy over time across the two conditions: two‐way mixed ANOVA, with experimental group (Learning, Movement) as between subjects factor, and time (blocks 1–6) as within‐subjects factor; there was no significant interaction (Experimental group × Time): *F*_(1.13,23.73)_ = 3.22, *p* = 0.081, partial η^2^ = 0.133 (Greenhouse–Geisser corrected). There was also no significant main effect of time on accuracy across both experimental groups: *F*_(1.13,23.73)_ = 3.85, *p* = 0.060, partial η^2^ = 0.155.

**Figure 3:**
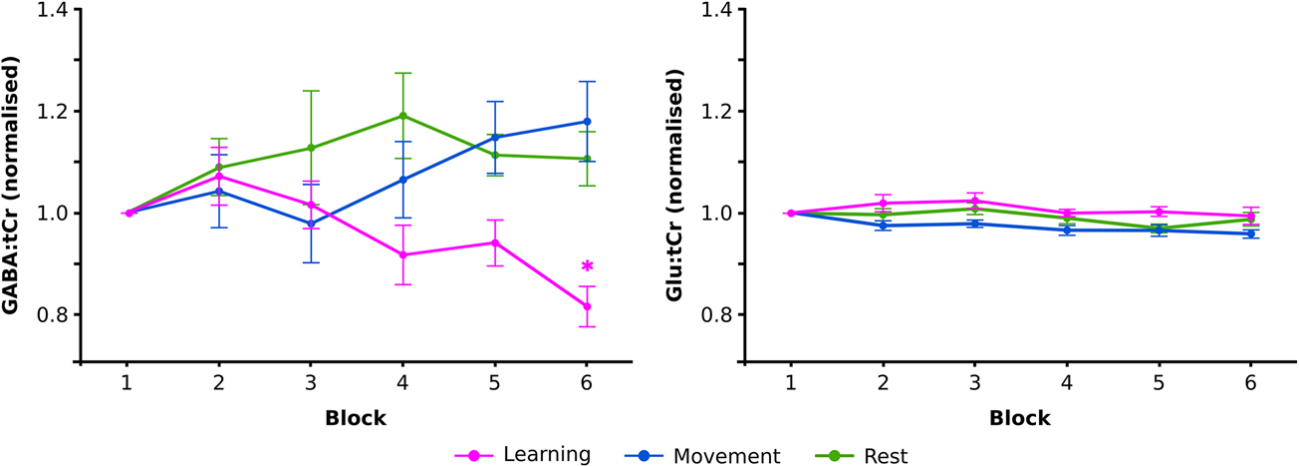
Motor learning is associated with a reduction in motor cortex GABA concentrations Group mean GABA:tCr and Glu:tCr concentrations presented normalised to block 1 for six serial MRS acquisitions measured during task performance. During motor sequence learning, a reduction in the concentration of motor cortex GABA:tCr is observed (pink) that is not seen in a motor task of equivalent duration lacking a learnable sequence (blue), nor during a passive resting task of the same duration (green). Two‐way mixed ANOVA with one factor of experimental group (Learning, Movement, or Rest) and one factor of block (1–6): *F*_(10,155)_ = 2.03, *p* = 0.034, partial η^2^ = 0.116. This effect was driven by a drop in GABA:tCr concentration in the Learning group (simple main effect of block: *F*_(5,50)_ = 4.16, *p* = 0.003, partial η^2^ = 0.294). Equivalent measures of glutamate showed no evidence of a change specific to the Learning group: a two‐way mixed ANOVA revealed no significant interaction between experimental group (Learning, Movement or Rest) and time on Glu:tCr: *F*_(10,155)_ = 0.780, *p* = 0.648, partial η^2^ = 0.048. ^*^ *p* < 0.05 Bonferroni‐adjusted *post hoc* pairwise comparison compared with block 1. Mixed ANOVAs were conducted using raw non‐normalised data; for visualisation purposes only, data were normalised to block 1. Within‐subject standard error bars were calculated across each group (Cousineau, 2005).

In the analysis of MRS GABA:tCr data, a two‐way mixed ANOVA revealed a significant interaction between experimental group (Learning, Movement or Rest) and time on GABA:tCr: *F*_(10,155)_ = 2.03, *p* = 0.034, partial η^2^ = 0.116, (Fig. 3) so simple main effects were analysed. In the Learning group there was a significant main effect of time on GABA:tCr: *F*_(5,50)_ = 4.16, *p* = 0.003, partial η^2^ = 0.294. *Post hoc* paired‐samples *t* tests revealed a significant reduction in GABA:tCr in block 6 compared with block 1 (*p* < 0.05, Bonferroni adjusted). There was no significant main effect of time on GABA:tCr in the Movement group (*F*_(5,55)_ = 1.16, *p* = 0.339, partial η^2^ = 0.096), nor in the Rest group (*F*_(5,50)_ = 0.226, *p* = 0.949, partial η^2^ = 0.022).

To investigate whether the observed decrease in GABA:tCr during learning might result from longitudinal changes in the fit or SNR of the MRS data specific to the Learning group we performed a two‐way mixed ANOVA on data quality and fit metrics from LCmodel, including: SNR, CRLB, FWHM and Fit values. These analyses revealed no significant interaction between experimental group (Learning, Movement or Rest) and time (Table 2).

### Early M1 GABA concentration is predictive of subsequent learning performance

**Figure 4:**
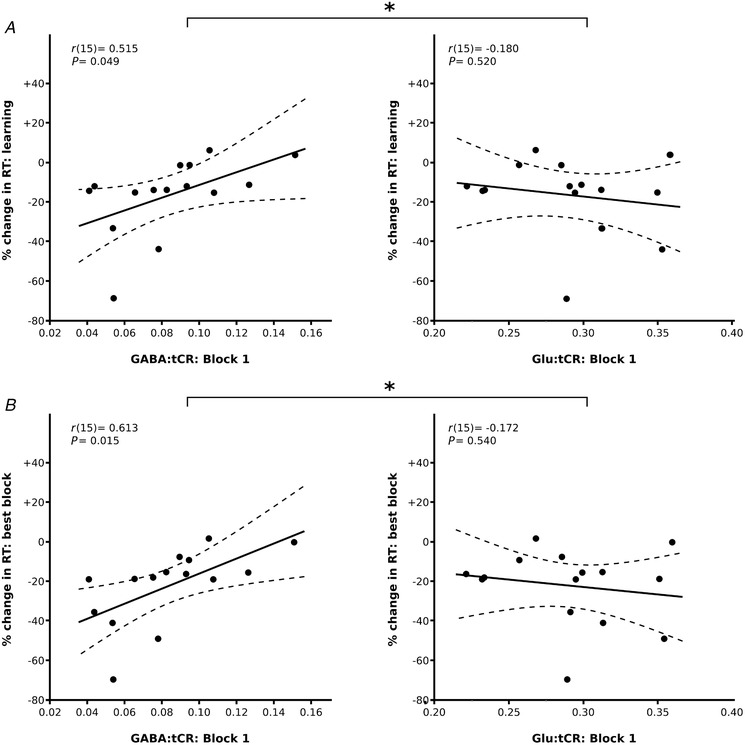
Early concentrations of GABA:tCr are correlated with the magnitude of subsequent motor learning *A*, a positive correlation was observed between the concentration of GABA:tCr in motor cortex and the reduction in response time due to learning, defined as the difference between median performance in sequence blocks 4–6 and sequence block 1, normalised to sequence block 1. *B*, early concentrations of GABA:tCr are also strongly correlated with the percentage change in reaction time in the best block, defined as the block (from blocks 2–6) in which the median reaction time was lowest. The same pattern was not observed with equivalent concentrations of excitatory glutamate (Glu:tCr). ^*^The correlation of GABA:tCr with learning and task performance differed significantly from equivalent correlations between Glu:tCr and the same behavioural measures: Hittner's *Z p* < 0.05.

We subsequently assessed whether levels of M1 inhibition early in learning predicted subsequent learning. This was investigated in the Learning cohort (*n* = 15), including additional participants whose GABA:tCr in block 1 met the CRLB quality criteria (<50%). There was a positive correlation between block 1 GABA:tCr and the percentage change in RT associated with learning (*r*(15) = 0.515, *p* = 0.049), such that lower levels of GABA:tCr in M1 early in the task are correlated with greater subsequent motor learning (Fig. 4 *A*). No such relationship was observed between the block 1 measure of M1 Glu:tCr and percentage change in RT (*r*(15) = −0.180, *p* = 0.520; Fig. 4 *A*). The correlation between M1 GABA:tCr and the percentage change in RT was significantly stronger than the equivalent relationship for Glu:tCr (Hittner's *Z* = 2.08, *p* = 0.038). The correlation between M1 GABA:tCr and the percentage change in RT persisted for GABA:tCr values uncorrected for voxel grey matter content, where the percentage grey matter was included as a covariate in a partial correlation: *r*(15) = 0.571, *p* = 0.033. There was no significant correlation between the RT in block 1 and the overall measure of learning (*r*(11) = 0.482; *p* = 0.07), but there was a trend suggesting people performing worse early in the task (block 1) went on to learn less overall. There was also no significant correlation between the magnitude of the change in GABA:tCr and the magnitude of learning (*r*(11) = −0.056; *p* > 0.88).

A secondary measure of learning, best block performance, captured the magnitude of learning in the block (from blocks 2 to 6) in which participants performed best, expressed relative to the first motor sequence block. There was a strong positive correlation between block 1 GABA:tCr and the best block performance (*r*(15) = 0.613, *p* = 0.015), such that lower levels of GABA:tCr in M1 early in the task are correlated with greater reduction in RT at peak performance (Fig. 4 *B*). No such relationship was observed between the block 1 measure of M1 Glu:tCr and best block performance (*r*(15) = −0.172, *p* = 0.540).

## Discussion

This study was performed to investigate the role of motor cortical GABA in human skill learning. We provide strong evidence for a reduction in the concentration of inhibitory GABA:tCr in M1 early during the acquisition of a learned sequence of movements. The observed reduction in GABA:tCr was specific to the movement observed in the Learning group: it was not observed in the context of the same finger movements, not associated with learning in the Movement group, nor observed in the Rest group which did not involve finger movements. Furthermore, the observed change in GABA concentration associated with motor learning was not mirrored by changes in glutamate concentrations also derived from MRS. The magnitude of motor learning in the Learning group also strongly correlated with the concentration of GABA:tCr in M1 early in the task. Taken together these results highlight the potentially crucial and early role for the disinhibition of M1 in supporting a learned movement.

Beyond the phasic GABAergic inhibition central to mechanisms such as lateral inhibition, the tonic activity from extracellular GABA is thought to mediate a basal level of inhibitory tone (Semyanov *et al*. 2004). This ambient inhibitory activity acts via extra‐synaptic GABA_A_ receptors, altering properties such as the membrane refractory period (Glykys & Mody, 2007; Belelli *et al*. 2009; Isaacson & Scanziani, 2011). Tonic signalling is thought to affect local network activity in a paracrine fashion: the GABAergic overspill from phasic GABA release leads to a tonic extracellular concentration of GABA, whose signalling impacts the excitability of neighbouring neurons (Farrant & Nusser, 2005). MRS measures of GABA concentration have been suggested to more closely represent the extracellular pool of GABA responsible for this tonic inhibitory activity, rather than the vesicular pool of GABA responsible for phasic signalling, which is bound to macromolecules and therefore may be less visible to MRS (Stagg *et al*. 2011 *b*,*c*). No correlations have been observed between MRS GABA and phasic GABA signalling using paired pulse TMS (Stagg *et al*. 2011 *a*; Dyke *et al*. 2017). However, a significant correlation between MRS GABA and tonic GABA has been observed (Stagg *et al*. 2011 *a*), although a more recent study has failed to replicate the relationship (Dyke *et al*. 2017). The metabolic GABA pool has no direct effect on neural signalling, although extracellular [GABA] is closely related to both metabolic and vesicular GABA levels. The MRS GABA signal is likely to be impacted by all these pools at least to some degree, although the specific combination of each has yet to be definitely determined. The results of this study are in line with previous TMS studies of motor learning, which suggest a role for both extra‐synaptic and synaptic GABA changes (Coxon *et al*. 2014).

Tonic inhibition is thought to result from the extrasynaptic overspill of GABA mediating phasic accumulation in the extracellular space of GABAergic activity. The evidence of decreased extracellular GABA during learning reported in this study is in keeping with observations of a reduced frequency of axonal boutons on inhibitory interneurons immediately after the initiation of training from murine studies of motor learning (Chen *et al*. 2015): a reduction in the phasic activity of GABAergic neurons may result in a reduction in the tonic extracellular GABA pool, which has a subsequent impact on the membrane refractory period and activity of local neurons. The widespread effect of network inhibition is in keeping with observations of heightened M1 excitability after learning (Muellbacher *et al*. 2001), potentially unmasking latent connections and facilitating plastic change in the connections within M1 (Huntley, 1997).

The correlation between M1 GABA in the earliest stages of task performance and the magnitude of subsequent motor learning (Fig. 4) is in keeping with the notion of M1 disinhibition acting as a precursor to the M1 plasticity associated with motor sequence learning. Specifically, greater levels of extracellular GABA acting tonically on local circuits in M1 may prevent or slow the process of local disinhibition associated with learning, such that the magnitude of learning observed in this study was less than that observed in participants whose extracellular GABA concentration was already comparatively low in the first MRS block. It has previously been demonstrated that the responsiveness of the GABAergic system is correlated with behavioural performance on a motor learning task (Stagg *et al*. 2011 *a*). It is therefore possible that the relationship between GABA and learning observed in this study could result from those individuals who had an early drop in GABA concentration subsequently learning more in the study, whereas those who maintain relatively high GABA concentrations early in the task go on to learn less. Our measure of early GABA:tCr encompasses the initial phase of learning, and therefore our data can only partially support this hypothesis.

The early concentrations of GABA:tCr in M1 were correlated with both the degree of learning (Fig. 4 *A*) and best block performance (Fig. 4 *B*). While motor learning measures consistently considered the relative difference between median RT in block 1 and blocks 4–6, the best block measure offered a potential measure of task performance that accounted for inter‐individual differences in the time course of learning across the duration of the task.

We observed no relationship between the magnitude of the reduction in M1 GABA and the change in RT as a measure of learning. However, we are cautious in drawing a firm conclusion from this null finding. The lack of a relationship between changes in GABA and changes in RT may reflect the fact that changes in the M1 concentration of GABA are only one aspect of learning; the GABAergic system interacts with the activity of a variety of other neuronal sub‐populations and cortical regions (Chen *et al*. 2015). Moreover, the observed reduction in GABA:tCr may not scale linearly with learning; learning may be contingent on a reduction in inhibition below a specific set point, beyond which greater disinhibition does not necessarily reflect greater learning. We also cannot rule out the possibility that the null result arose from a limitation of the MRS method, particularly the limited temporal resolution of the GABA:tCr measurements. It is also highly likely that the dynamics of the GABA:tCr signal would continue to evolve beyond the 40 min period of measurement. The time constraint here represents the practical feasibility of acquiring high‐quality spectra over prolonged continuous periods. Further work focused on understanding the dynamics of the GABA signal during learning could potentially overcome these limitations, and may reveal a relationship between GABA change and learning. An alternative explanation for the observed reduction in GABA:tCr could be a metabolic change in M1 during learning, such as increased GABA catabolism via the tricarboxylic acid (TCA) cycle. However, the relative stability of measured glutamate concentrations and the lack of a change in GABA:tCr during a matched movement condition provide evidence to mitigate this possibility.

No change in the MRS concentration of glutamate was observed alongside the reduction of GABA in the context of motor learning, movement or rest. Glutamatergic signalling encompasses a broad range of processes in the cerebral cortex; our results do not exclude the possibility of a change in glutamatergic signalling associated with learning, but rather suggest that our quantification of glutamate may represent a composite of its various roles as both a neurotransmitter and a metabolite. The application of short echo time MRS acquisitions in this study may also have limited the ability to measure changes in the glutamatergic system due to the predominance of a signals from restricted vesicular pools, reducing sensitivity to change (Mullins, 2018). In addition, MRS measures are also not able to quantify changes in glutamate receptor density, which could impact its signalling across the course of learning.

We observed a specific reduction in GABA during motor learning; no change in GABA was observed during simple movement. This finding is consistent with the one previous study highlighting reduced MRS‐assessed GABA concentrations in human M1 observed during a force‐tracking learning task but not in an analogous movement condition (Floyer‐Lea, 2006). However, these results are in contrast to a recent study reporting evidence of a reduction in MRS measures of GABA during a bi‐manual whole‐hand clench task (Chen *et al*. 2017). In light of differences in the relative balance of left and right M1 in the context of unimanual *versus* bimanual tasks (Koeneke *et al*. 2004), it is difficult to interpret the present results in the context of this study, where the concentration of M1 GABA change may be impacted by a mixture of top‐down signals and M1–M1 interhemispheric signals that differs from those occurring in a unimanual task.

This work represents an important replication and extension of previous findings regarding the role of M1 inhibition in motor learning. We provide strong evidence for a learning‐specific reduction in the measured concentration of M1 GABA, likely to represent a change in the level of local inhibitory tone affected by extrasynaptic GABAergic signalling. Further, we demonstrate a cross‐sectional correlative relationship between the concentration of M1 GABA at an early time point in the task and the magnitude of subsequent motor learning, providing initial support for a potential causal link between the set point of local inhibitory tone and the propensity for subsequent plastic change to support behavioural change. Taken together these findings suggest that alterations in inhibitory signalling in M1 probably represent an important step in the mechanism of plasticity that supports motor learning. This work again highlights the potential for MRS to quantify changes in neurochemicals such as GABA over time in the context of a specific task or exposure.

## Additional information

### Competing interests

The authors declare no competing interests in relation to this work.

### Author contributions

Conception and design of the work (JK, ELH, CJS). Data acquisition, analysis and interpretation (JK, ELH, APDZ, AR, UEE, CJS). Drafting and revising the manuscript (JK, ELH, APDZ, AR, UEE, CJS). All authors approved the final version of the manuscript and agree to be accountable for all aspects of the work. All individuals who qualify for authorship are listed herein.

### Funding

JK holds a Wellcome Trust Sir Henry Wellcome Postdoctoral Fellowship (204696/Z/16/Z) and was also supported by a Stevenson Junior Research Fellowship at University College (Oxford) during this work. CJS holds a Wellcome Trust/Royal Society Sir Henry Dale Fellowship (102584/Z/13/Z). ELH was additionally supported by the NIHR Oxford Biomedical Research Centre. Support for the 7 T scanner was provided by the Medical Research Council. The Wellcome Centre for Integrative Neuroimaging is supported by core funding from the Wellcome Trust (203139/Z/16/Z).
